# Differences in Cumulative Long-Term Care Costs by Community Activities and Employment: A Prospective Follow-Up Study of Older Japanese Adults

**DOI:** 10.3390/ijerph18105414

**Published:** 2021-05-19

**Authors:** Masashige Saito, Naoki Kondo, Jun Aida, Junko Saito, Hisataka Anezaki, Toshiyuki Ojima, Katsunori Kondo

**Affiliations:** 1 Faculty of Social Welfare, Nihon Fukushi University, Aichi 470-3295, Japan; 2 Center for Well-Being and Society, Nihon Fukushi University, Aichi 460-0012, Japan; 3 Department of Social Epidemiology and Global Health, Graduate School of Medicine and School of Public Health, Kyoto University, Kyoto 606-8501, Japan; kondo.naoki.0s@kyoto-u.ac.jp; 4 Department of Oral Health Promotion, Tokyo Medical and Dental University, Tokyo 113-8510, Japan; aida.ohp@tmd.ac.jp; 5 Center for Public Health Science, National Cancer Center Japan, Tokyo 104-0045, Japan; j.junkosaito@gmail.com; 6 National Cerebral and Cardiovascular Center, Osaka 564-8565, Japan; hisataka.anezaki@ncvc.go.jp; 7 Department of Community Health and Preventive Medicine, Hamamatsu University School of Medicine, Shizuoka 431-3192, Japan; ojima@hama-med.ac.jp; 8 Center for Preventive Medical Sciences, Chiba University, Chiba 260-0856, Japan; kkondo@kkondo.net; 9 Center for Gerontology and Social Science, National Center for Geriatrics and Gerontology, Aichi 474-8511, Japan

**Keywords:** public long-term care insurance, cumulative cost, community activities, employment, older adults

## Abstract

We evaluated differences in the cumulative benefit costs of public long-term care (LTC) insurance services by employment status and frequency of community activities. A baseline survey was conducted on functionally independent older people from 12 municipalities as a nationwide survey from 2010 to 2011. Employment status was dichotomized, and community activity was assessed based on the frequency of participation in hobbies, sports clubs, or volunteering. We followed the respondents’ LTC service costs over a period of 6 years using public LTC claim records (*n* = 46,616). We adopted a classical linear regression analysis and an inverse probability weighting estimation with multiple imputation for missing values. Compared with non-participation in each community activity, the cumulative LTC costs among individuals who participated in hobbies or sports group activities at least twice a week were 1.23 (95% confidence interval: 0.73–1.72) to 1.18 (0.68–1.67) thousand USD lower per person over the 6-year period (28.7% to 30.1% lower, respectively). Similarly, the costs for employed persons were 0.55 (0.20–0.90) to 0.64 (0.29–0.99) thousand USD per person lower than among retirees (14.5% to 16.9% lower). Promoting employment opportunities and frequent participation in community activities among older adults may help reduce future LTC costs by around 20% as a result of extending healthy longevity.

## 1. Introduction

One of the most important issues faced by super-aged societies is the expansion of costs related to long-term care (LTC). Among all countries, Japan is experiencing the highest rate of aging. The proportion of older people is 27.7% (as of 2017), and is predicted to reach around 40% by 2065 [1]. The Japanese public LTC insurance system launched in 2000, and since that time, has been confronted with the rapid expansion of costs. These costs are expected to more than double, from 100 billion USD in 2016 to 210 billion USD by 2025. The latter costs are expected to account for about 13% of the social security benefit in Japan [2].

To reduce LTC insurance costs for sustainable management, the policy of the Japanese government has involved the promotion of diverse types of social participation among older adults. According to activity theory [3] and productive aging [4], participating in employment and community activities provides more opportunities to acquire social roles and contacts, build self-esteem, self-efficacy, and morale, and facilitate access to various types of information leading to healthy and successful aging. Several systematic reviews have reported the physical, psychological, and social benefits of social participation among older people as a social determinant of health [5,6,7]. Previous studies involving older Japanese adults have shown that participating in collective activities such as hobbies, sports clubs, or volunteering in the local community lowers the incidence of functional disabilities [8,9,10,11,12,13], functional decline [14,15,16,17], and cognitive decline or dementia [18,19]. Employment is also considered to be a form of social participation that confers health benefits because retirement has been shown to worsen both mental health and high-level functional capacity [20].

We hypothesize that if healthy longevity could be extended and care requirements and support needs could be reduced through social participation, then the cumulative cost of LTC insurance services would be reduced. One recent Japanese study reported that participating in hobbies or sports groups at least once a week was negatively associated with the cumulative cost of LTC insurance services, with reductions of approximately 3500–6000 USD per person over an 11-year period [21]. However, the result lacked generalizability, because that study was based on only one municipality. In Japan, the insurers in the LTC system are municipalities or special wards in the area. The insurance premium for older persons and the supply of LTC services differ remarkably in each municipality to reflect the specific characteristics and budget of each area. In addition, no evidence of an association between employment status and LTC costs has been presented. Therefore, in this study, we aimed to assess differences in the cumulative cost of public LTC insurance services by social participation, including employment, based on governmental claims records with a questionnaire survey conducted on older Japanese adults in multiple municipalities.

## 2. Materials and Methods

### 2.1. Study Setting

**Figure 1:**
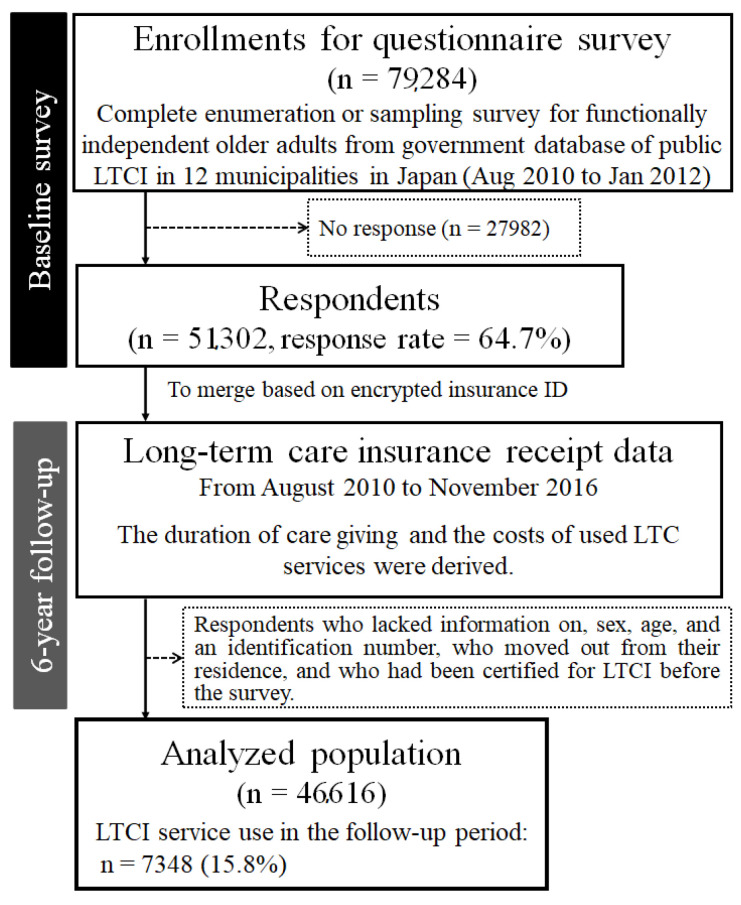
Flow diagram of the analyzed population.

We conducted a large-scale nationwide prospective cohort study. Baseline data were collected through a self-administered questionnaire from August 2010 to December 2011 as part of the Japan Gerontological Evaluation Study (JAGES). We obtained 51,302 individuals living in 12 municipalities of Japan who were aged 65 years or older, physically and cognitively independent, and living independently in the community (response rate = 64.7%). The municipalities were not randomly selected but covered a wide range of characteristics in terms of region and population size in Japan. The study areas included both urban and rural area in Hokkaido, Chiba, Yamanashi, Aichi, Mie, and Nagasaki prefectures, with a densely inhabited district ranging from 32.8 to 7286.6 inhabitants per square kilometer (average in Japan: 1100.0 people/km^2^), and the proportion of older adults ranging from 22.8% to 36.3%. In nine relatively small municipalities, the questionnaire was performed by a complete enumeration, and in three municipalities, random sampling was adopted. Next, we obtained claim records from a governmental database regarding public LTC insurance benefits over a period of 6 years from the baseline survey. After eliminating those who lacked information on sex and age or who had moved out of their residence, 46,616 respondents (follow-up rate = 90.9%) were linked to the public LTC claim records (Figure 1).

### 2.2. Ethical Approval

This study was performed through a collaborative research agreement with the associated municipalities. Ethical approval (No. 2493) was provided by the Ethics Board at Chiba University. JAGES participants were informed that participation in the study was voluntary and that completing and returning the questionnaire via mail indicated their consent to participate in the study. We created an anonymized dataset. All methods were carried out in accordance with relevant guidelines and regulations or in accordance with the Declaration of Helsinki.

### 2.3. Measurement of the Cumulative Long-Term Care Costs

The primary outcome of the study was the cumulative cost of public LTC insurance services during the follow-up period. We summed costs for every month over the follow-up period because seasonal bias in the use of LTC services has often been reported [22]. In Japan, public LTC insurance services are available only to people who have certified care needs (levels 1 to 5) or support needs (levels 1 or 2). This study is based on a prospective cohort study. The LTC costs in all participants are zero in the first month, because we investigated the people who were physically and cognitively independent (that is, LTC non-users) at the time of baseline survey. The cumulative cost is zero in the following cases: deceased individuals who did not have certified care needs or support needs, and non-service users in follow-up period. The currency exchange rate used in the present study was 100 JPY = 1 USD. Since the consumer price index regarding medical care and welfare, and the long-term care costs per recipient were almost unchanged, the inflation is almost non-existent in this period.

The Japanese LTC system operates with a basis of social insurance principles. Only services are provided, not cash allowances, and recipients can choose their own services and providers. The public LTC claim record does not include costs not covered by insurance, such as food, housing, and childcare expenses. In general, 10% of these costs are co-payments. For more information concerning the LTC insurance system in Japan, please see Tsutsui et al. [23].

### 2.4. Community Activities and Employment Status

We focused on the major three types of community activities previously identified as being associated with a lower risk for functional disabilities: participation in a hobby activities group [9], a sports group or club [8,9], and a volunteer group [10,11]. Respondents were asked how often they took part in these activities by rating the frequency of participation on a five-point scale (never, a few times a year, once or twice a month, once a week, and twice a week or more). We then compared differences in the frequency of participation in each community activity. Employment status was dichotomized as follows based on current work status: employed or retired/never had a job.

### 2.5. Covariates

Demographic variables as potential confounders included sex, age, years of education, equivalent income, marital status, and living situation. These variables have been associated with healthcare service utilization in previous studies [24,25,26,27]. These variables are also basic variables as social determinants of health. Age was categorized as follows: 65–74, 75–84, and 85+ years. Years of education was categorized as <6, 6–9, 10–12, and 13+. We equivalized household income by the square root of the numbers and classified it as <20.0, 20.0–29.9, 30.0–39.9, and 40.0+ thousand USD. Marital status consisted of married, widowed, divorced, and never married. Living situation was dichotomized into living alone or not. To account for health status at baseline, presence of disease or impairment, recognition of forgetfulness, and self-rated health were considered. Presence of disease or impairment was based on self-reported medical condition and classified as: no illness, having illness but not needing treatment, having illness but discontinued treatment, and receiving some treatment. We also dichotomized this variable as: none or present. Recognition of forgetfulness was also dichotomized as: none or present. We assessed self-rated health using the following four categories: excellent, good, fair, and poor. Finally, to consider access to LTC services, we controlled for the municipality of residence as a dummy variable. The distributions of these covariates are shown in supplementary materials (Table S1). The people designated as female, old-old, having low socio-economic status, and being in poor health were higher in cumulative cost of LTC services in this follow-up period.

### 2.6. Statistical Analysis

After calculating the descriptive statistics, we conducted two regression analyses. First, we adopted a classical linear regression (ordinary linear squares [OLS]) model, controlling for the covariates at baseline. Employment status and each community activity were inputted in same model, because the aim and characteristics of activity is remarkably different. In this model, all data for an observation that had one or more missing values were removed for a complete case analysis. Second, we performed a multiple imputation technique by chained equations under the missing at random assumption, which means that systematic differences may be present between the missing and observed values. We created 20 imputed datasets. After that, to control for potential unmeasured confounders, we adopted the inverse probability weighting (IPW) estimator [28,29] using the imputed datasets. We calculated the generalized propensity scores using multinomial regression analysis and the C-statistic, employing all previously listed covariates. We carried out the following for a robustness check. Assuming that the distribution of LTC costs was censored at zero for a large portion of the sample, we estimated a regression equation using the Tobit model. Previous research suggested that time to death is a strong predictor of higher medical and LTC expenditure among the older adults [30,31]. To exclude the fluctuation effects immediately before death, we analyzed only survival cases during the follow-up period and the average monthly LTC costs during survival. Finally, as a rough simulation, we calculated the difference between current costs and lowest case costs, assuming that all people fell under the condition involving the most cost-saving category. All statistical analyses were carried out using Stata 15.1 (StataCorp, College Station, TX, USA).

## 3. Results

Table 1 shows the descriptive statistics of community activities and employment status among the respondents. Cumulative LTC costs and mortality rates were remarkably lower in employed persons and participants in hobbies, sports, and volunteering groups than in retirees and nonparticipants. For instance, the mean LTC cost was 4.28 thousand USD among nonparticipants in hobby activities, compared with 1.86 thousand USD in people who participated in hobby activities at least twice a week.

The OLS model showed that, compared with non-participation in each community activity and retirees/never had a job, respondents who were employed and participated in community activities had a lower LTC cumulative costs, even after controlling for covariates (Table 2). The IPW estimations with multiple imputed datasets showed similar trends. The LTC costs among individuals who participated in hobbies or sports group activities at least twice a week were approximately 1.23 thousand USD (95% confidence interval [CI]: 0.73–1.72) or 1.18 thousand USD (95% CI: 0.68–1.67) lower per person compared with nonparticipants in each activity. Similar trends were also confirmed in people who participated once a week. By contrast, participation in volunteer groups showed different trends. No significant difference was observed for the more frequent group, but LTC costs among people who had participated a few times a year and once or twice a month were 0.70 thousand USD (95%CI: 0.10–1.30) and 0.81 thousand USD (95% CI: 0.23–1.40) lower, respectively. Each model showed that the cumulative LTC costs was approximately 0.6 (0.55 to 0.64) thousand USD per person lower among employed persons than among retirees. The covariate balance summary and C-statistics for these IPW estimations are shown in Supplementary Materials (Tables S2 and S3). When we changed the estimation method to the Torbit model and analyzed only survival cases and average monthly LTC costs during survival, the direction of the association did not largely change (see Supplementary Materials, Table S3).

The cumulative LTC costs among people who participated in hobby or sports group activities at least twice a week were about 30% lower compared with nonparticipants (Table 3). Likewise, the cumulative LTC costs of less frequent participants in volunteer activities and employed persons were about 20% and 17% lower, respectively. Assuming that all people fell under the condition involving the most cost-saving category, promoting participation in hobbies or sports activities at least twice a week could be expected to contribute about a 20–25% reduction in LTC costs.

## 4. Discussion

To the best of our knowledge, this is the most recent article to demonstrate that promoting community activities and employment among older adults might help lower subsequent LTC costs in Japan. The main findings are as follows: (1) the cumulative LTC costs among respondents who actively participated in hobbies and/or sports group activities was lower about 30% compared to nonparticipants; (2) the cumulative LTC costs among employed people was also about 17% lower than that among retirees/never had a job; and (3) moderate participation in volunteer activities was associated with lower cumulative LTC costs. An incentivization scheme for government office, companies, and investors, such as social impact bonds and “pay for success” programs, are being promoted globally. The evidence regarding the financial benefit has important implications for LTC and public health in regard to providing more community activities or job opportunities for older adults.

These findings support the preceding theory in social gerontology [3,4] that promoting diverse social participation can contribute to healthy and successful aging. This study adds evidence suggesting that active participation in community activities may be effective for not only preventing functional deterioration, but also reducing LTC costs. In addition, concerning the hobby activities group, low frequent participation was also significantly associated with a reduction in cumulative LTC service costs, which might reflect that participation in hobbies is mainly associated with pleasure. According to our result, a relatively lower proportion of older adults were found to have never participated in hobbies compared with sports activity (54.1% vs. 72.8%, respectively). Among older adults, those who do not have a hobby might be at a high risk for poor health. Simultaneously, it is also important to note that the point estimations of all frequencies in hobbies and sports group activities were in same direction, even if some were not statistically significant. In recent years, around 60% of older Japanese adults have participated in community activities, including sports clubs, hobby activities, and regional events [32]. The impact of about a 20–25% reduction in LTC costs cannot be ignored.

This trend is roughly consistent with that reported in a previous study, which was based on relatively weak statistical power with a small sample [21]. The high generalizability of the present results was confirmed owing to the large-scale data from multiple municipalities. Differences in LTC costs may have arisen because of increases in healthy longevity or reductions in the period of functional disability, rather than restrictions on the use of the required services. Moreover, our findings might be an underestimation because the less frequent categories for each community activity tended to be associated with higher mortality rates. These values may have been lower among people who died earlier because we analyzed cumulative LTC costs during the follow-up period. In addition, the results from only survival cases and for average monthly LTC costs during survival did not largely change. These findings suggest that the fluctuation in LTC costs immediately before death is relatively small.

Our results showed that employment status contributed to reductions in cumulative LTC costs, although diverse explanations about the health effects of retirement have been presented [33]. Compared with community activities, the difference in LTC costs by employment status was relatively small; this may have been the result of our limited indicator in terms of not being able to measure the frequency of work. The frequency of work among employed persons would be relatively low because most older Japanese workers are employed as non-regular employees [34]. The Japanese government has set an agenda to promote the dynamic engagement of all citizens. Considering the high labor force participation rate among older Japanese adults [34,35], promoting employment opportunities and community activities among older adults might have a cost-containment effect for LTC costs in Japan that cannot be ignored.

On the other hand, for volunteer activities, less frequent participation was associated with lower LTC costs; this finding is also consistent with a previous report [21]. It has often been mentioned that volunteers, including local welfare commissioners, tend to experience excessive burdens, since most of them shoulder substantial responsibilities and work without receiving any pay [36,37]. Our results suggest that managing volunteer activities that carry only a moderate burden and frequency would contribute to not only an extension of healthy longevity, but also a reduction in LTC costs.

The strength of this study is that we analyzed merged individual data from questionnaires on social life and public claim records as they pertain to LTC services. More specifically, we used a large-scale dataset involving data from numerous municipalities. However, this study did have several limitations. First, there is a possibility of selection bias at the municipal level because our data were obtained from non-nationally representative samples. Second, we assessed the frequency of community activities, employment status, and covariates only at baseline. Therefore, we cannot examine causal relationships, even though we also controlled for multiple health dimensions and other covariates through the use of several statistical techniques. Third, since the baseline survey was based on a self-report questionnaire, a measurement bias cannot be ruled out. However, it is important to note that no activity discussed in this paper is special and unique; each activity is already common and can be assessed using a questionnaire survey. Fourth, although we focused on employment status as social participation in a broad sense, we did not assess the information in detail. Fifth, the follow-up period (6 years) was relatively short. The percentage of people who had certified care or support needs during the follow-up period was low, at most a little above 20%. A recent study has mentioned the phenomenon of “rejuvenation” among the new generation of Japanese older adults [38]. To estimate differences in lifelong LTC costs, future studies should use a longer follow-up period.

## 5. Conclusions

Community activities and employment status among older adults were negatively associated with the cumulative cost of LTC services. This suggests that promoting employment opportunities and participation in community activities among older adults may help reduce future LTC costs as a result of extending healthy longevity.

## Figures and Tables

**Table 1:** Descriptive statistics of cumulative LTC service costs.

			Cumulative Cost in LTC Services (Thousand USD)	Onset of Care/Support Needs	Mortality
	*n* ^1^	%	Mean ± SD	%	%
***Community activities***					
**Hobby activities group**					
Never	20,265	54.1	4.28 ± 16.86	23.6	13.9
A few times a year	3261	8.7	2.14 ± 10.86	14.9	9.4
Once or twice a month	5221	13.9	2.07 ± 10.80	15.6	8.0
Once a week	4301	11.5	2.25 ± 11.13	16.7	7.0
Twice a week	4411	11.8	1.86 ± 10.81	14.5	7.6
**Sports group or club**					
Never	26,286	72.8	3.92 ± 15.99	22.5	13.0
A few times a year	1477	4.1	1.72 ± 10.50	13.2	8.7
Once or twice a month	1612	4.5	1.86 ± 11.01	11.4	8.0
Once a week	2562	7.1	1.59 ± 9.02	13.3	5.3
Twice a week	4183	11.6	1.55 ± 9.73	13.0	6.5
**Volunteer group**					
Never	28,128	82.2	3.58 ± 15.35	20.7	12.0
A few times a year	2353	6.9	1.55 ± 8.95	12.4	8.4
Once or twice a month	2031	5.9	1.43 ± 8.89	12.2	6.1
Once a week	875	2.6	1.72 ± 10.47	12.6	7.8
Twice a week	836	2.4	2.05 ± 10.73	14.0	7.1
***Employment status***					
Retired/never had a job	30,706	76.1	3.79 ± 15.45	22.7	12.6
Employed	9625	23.9	1.48 ± 9.28	10.9	7.8

1 USD ≈ 100 JPY. ^1^ The total number of cases in each variable are not coincident, due to removing the observation that has missing value.

**Table 2:** Differences in cumulative LTC service costs over the 6-year follow-up period by community activities and employment status.

			Type of Community Activities			
	Hobby Activities Group		Sports Group or Club		Volunteer Group	
	OLS ^1,2^	IPW with MI ^3,4^	OLS ^1,2^	IPW with MI ^3,4^	OLS ^1,2^	IPW with MI ^3,4^
**Freq. of community activities**						
Never	ref.	ref.	ref.	ref.	ref.	ref.
A few times a year	−0.86 *** (−1.31 to −0.41)	−0.71 * (−1.29 to −0.12)	−0.28 (−0.92 to 0.37)	−0.39 (−1.14 to 0.36)	−0.90 *** (−1.29 to −0.51)	−0.70 * (−1.30 to −0.10)
Once or twice a month	−0.85 *** (−1.26 to −0.44)	−1.11 *** (−1.57 to −0.67)	−0.20 (−0.87 to 0.48)	−0.42 (−1.15 to 0.30)	−0.72 ** (−1.18 to −0.25)	−0.81 ** (−1.40 to −0.23)
Once a week	−0.91 *** (−1.33 to −0.48)	−1.01 *** (−1.51 to −0.51)	−0.94 *** (−1.33 to −0.56)	−1.14 *** (−1.71 to −0.57)	−0.10 (−1.12 to 0.91)	−0.51 (−1.47 to 0.46)
Twice a week †	−0.81 ** (−1.32 to −0.29)	−1.23 *** (−1.72 to −0.73)	−0.71 ** (−1.15 to −0.27)	−1.18 *** (−1.67 to −0.68)	−0.49 (−1.25 to 0.26)	−0.18 (−1.07 to 0.71)
**Employment status**						
Retired/never had a job	ref.	ref.	ref.	ref.	ref.	ref.
Employed	−0.52 ** (−0.81 to −0.22)	−0.64 *** (−0.99 to −0.29)	−0.57 *** (−0.86 to −0.28)	−0.63 *** (−0.98 to −0.28)	−0.47 ** (−0.76 to −0.18)	−0.55 ** (−0.90 to −0.20)

*** *p* < 0.001 ** *p* < 0.01 * *p* < 0.05 † *p* < 0.10 Unit: thousand USD (95% confidential interval) thousand USD ≈ 100 thousand JPY. OLS: Ordinary least squares regression. IPW: Inverse probability weighting. MI: Multiple imputation. ^1^ These estimations were from observations that had one or more missing values removed (complete case analysis). ^2^ The results were controlled by sex, age, years of education, equivalent income, marital status, living situation, disease and/or impairment, recognition of forgetfulness, self-rated health, and municipality at baseline. ^3^ Multiple imputation by chained equations was performed using sex, age, years of education, equivalent income, marital status, living situation, disease and/or impairment, recognition of forgetfulness, self-rated health, employment, and municipality (m = 20). ^4^ The generalized propensity scores were calculated using multinominal regression analysis using all previously listed potential confounders: sex, age, years of education, equivalent income, marital status, living situation, disease and/or impairment, recognition of forgetfulness, self-rated health, and municipality.

**Table 3:** Proportion of differences in cumulative LTC costs by community activities and employment status.

	N% [A]	Current Costs Difference			Difference from Lowest Case		
		Estimation ^1^ (Thousand $)	Mean (Thousand $) [B] ^3^	Prop. of Difference ^4^	Current Average Cost [C] ^5^	Lowest Case [D] ^6^	Difference (%) ^7^
**Hobby activities group**							
Never	54.1%	ref.	4.28	ref.	2.32	1.65	
A few times a year	8.7%	−0.71	3.57	−16.6%	0.31	0.27	
Once or twice a month	13.9%	−1.11	3.17	−25.9%	0.44	0.42	
Once a week	11.5%	−1.01	3.27	−23.6%	0.38	0.35	
Twice a week	11.8%	−1.23	3.05	−28.7%	0.36	0.36	
Total					3.81	3.05	−19.9%
**Sports group or club**							
Never	72.8%	ref.	3.92	ref.	2.85	1.99	
A few times a year	4.1%	−0.39	3.53	−9.9%	0.14	0.11	
Once or twice a month	4.5%	−0.42	3.50	−11.0%	0.16	0.12	
Once a week	7.1%	−1.14	2.78	−29.1%	0.20	0.19	
Twice a week	11.6%	−1.18	2.74	−30.1%	0.32	0.32	
Total					3.67	2.74	−25.3%
**Volunteer group**							
Never	82.2%	ref.	3.58	ref.	2.94	2.33	
A few times a year	6.9%	−0.70	2.88	−19.6%	0.20	0.19	
Once or twice a month	5.9%	−0.81	2.77	−22.6%	0.16	0.16	
Once a week	2.6%	−0.51	3.07	−14.0%	0.08	0.07	
Twice a week	2.4%	−0.18	3.40	−5.0%	0.08	0.07	
Total					3.47	2.77	−20.1%
**Employment status**							
Retired/never had a job	76.1%	ref.	3.79	ref.	2.88	2.40	
Employed	23.9%	−0.64 ^2^	3.15	−16.9%	0.75	0.75	
Total					3.64	3.15	−13.4%

^1^ Data of the estimations were from inverse probability weighting with multiple imputation (IPW with MI) model in Table 2. ^2^ This estimation (−0.64) is based on upper result from three IPW with MI models; lower result is −0.55. ^3^ These descriptive statistics were derived from former estimations with the mean of the cumulative cost (thousand USD) in the reference category, which was shown in Table 1: Hobby activities group = 4.28, sports group or club = 3.92, volunteer group = 3.58, and employment status = 3.79. ^4^ We calculated the proportion of each estimation to the mean of the cumulative cost (thousand USD) in the reference category. ^5^ Current average cost was calculated by [A] × [B]. ^6^ Average cost in the lowest case was calculated by [A] × minimum value of [B]: Hobby activities group = 3.05, sports group or club = 2.74, volunteer group = 2.77, and employment status = 3.15. This cost assumed that all people belonged to the the most cost-saving category. ^7^ This was calculated by the following formula; ([D]/[C]) − 1.

## Data Availability

The data are not publicly available because they contain information that could compromise the privacy of the research participants. The data that support the findings of this study are available on request from qualified researchers by e-mailing the data management committee: dataadmin@jages.net.

## Supplementary Materials

The following are available online at https://www.mdpi.com/article/10.3390/ijerph18105414/s1, Table S1: Characteristics of the survey respondents at baseline, Table S2: Covariate balance summary: standardized differences, Table S3: C-statistics using a multinomial regression model, Table S4: Robustness checks examining the differences in cumulative LTC service cost by community activities and employment status.
