# Supplementary material for: Differences in Cumulative Long-Term Care Costs by Community Activities and Employment: A Prospective Follow-Up Study of Older Japanese Adults
Source: Int J Environ Res Public Health. 2021 May 19;18(10):5414. doi: 10.3390/ijerph18105414 (PMC8158700; doi:10.3390/ijerph18105414)
Supplement: Supplementary file 1 [file ijerph-18-05414-s001.zip › 9_Supplementary Material 1r.pdf]

Table S1. Characteristics of the survey respondents at baseline [1]

	Total		Cumulative cost of LTC services in 6 years
	n[2]	%	(thousand USD)[3] Mean ± SD
Sex			
Female	25031	53.7	4.31 ± 16.83
Male	21585	46.3	2.76 ± 12.56
Age			
65-74	27000	57.9	1.11 ± 8.31
75-84	16675	35.8	5.47 ± 18.09
85+	2941	6.3	15.69 ± 29.41
Years of education			
6-9	22235	49.1	4.20 ± 16.38
10-12	15182	33.5	2.84 ± 13.01
13+	7889	17.4	2.45 ± 12.12
Equivalent income (1000USD)			
- 19.9	13236	41.6	3.16 ± 14.13
20.0 - 29.9	8886	27.9	3.06 ± 13.95
30.0 - 39.9	5576	17.5	2.37 ± 12.03
40.0+	4117	12.9	2.38 ± 11.69
Marital status			
Married	32764	72.1	2.51 ± 12.29
Widowed	10087	22.2	6.83 ± 20.85
Divorced	1605	3.5	2.49 ± 11.31
Never married	992	2.2	5.00 ± 18.68
Living situation			
Living alone	6163	13.5	5.36 ± 18.63
Living with others	39644	86.5	3.25 ± 14.18
Disease and/or impairment			
None	10360	24.9	2.44 ± 12.84
Presence	31294	75.1	3.79 ± 15.43
Recognition of forgetfulness			
None	36058	82.4	2.86 ± 13.30
Presence	7720	17.6	6.54 ± 20.31
Self-rated health			
Excellent	5333	11.7	1.65 ± 10.03
Good	30926	67.9	2.91 ± 13.25
Fair	7848	17.2	6.23 ± 19.76
Poor	1426	3.1	9.12 ± 25.19

1. This dataset was composed twelve municipalities. Composition ratio of respondents was as follows: A town=2.5%, B town=2.7%, C town=4.4%, D city=6.4%, E city=7.1%, F city=29.7%, G city=7.7%, H city=13.9%, I town=5.8%, J town=9.0%, K town=3.0%, L city=7.7%
2. The observations that had missing values were removed.
3. 1USD≈100JPY
