# Supplementary material for: Differences in Cumulative Long-Term Care Costs by Community Activities and Employment: A Prospective Follow-Up Study of Older Japanese Adults
Source: Int J Environ Res Public Health. 2021 May 19;18(10):5414. doi: 10.3390/ijerph18105414 (PMC8158700; doi:10.3390/ijerph18105414)
Supplement: Supplementary file 1 [file ijerph-18-05414-s001.zip › 9_Supplementary Material 2.pdf]

Table S2. Covariate balance summary: standardized differences

	Hobby activities group (ref. never)							
	Twice a week +		Once a week		Once or twice a month		A few times a year	
	Raw	Weighted	Raw	Weighted	Raw	Weighted	Raw	Weighted
Sex	-0.162	0.027	-0.328	-0.013	-0.140	-0.004	0.263	0.001
Age	-0.161	-0.005	-0.153	0.006	-0.156	-0.013	-0.171	-0.002
Years of education	0.220	-0.015	0.322	0.007	0.337	0.008	0.256	0.012
Equivalent income	0.111	-0.009	0.197	-0.002	0.198	0.009	0.136	-0.007
Marital status	-0.073	-0.005	-0.014	-0.007	-0.063	-0.005	-0.105	0.016
Living situation	0.073	-0.004	0.063	0.000	0.044	-0.007	-0.026	0.016
Disease and/or impairment	0.062	-0.019	0.028	0.012	0.031	0.010	0.036	-0.006
Recognition of forgetfulness	-0.152	0.001	-0.115	0.013	-0.136	-0.005	-0.117	-0.010
Self-rated health	-0.392	0.018	-0.319	-0.021	-0.301	-0.006	-0.212	-0.005
Municipality	-0.037	0.008	-0.060	-0.009	-0.036	-0.011	-0.102	0.007
	Sports group or club (ref. never)							
	Twice a week +		Once a week		Once or twice a month		A few times a year	
	Raw	Weighted	Raw	Weighted	Raw	Weighted	Raw	Weighted
Sex	-0.068	0.024	-0.268	0.017	0.307	-0.001	0.476	-0.012
Age	-0.187	-0.003	-0.251	-0.003	-0.226	-0.012	-0.210	-0.006
Years of education	0.208	-0.009	0.311	0.012	0.388	0.026	0.274	-0.031
Equivalent income	0.086	-0.002	0.213	0.012	0.279	-0.016	0.139	-0.054
Marital status	-0.131	-0.015	-0.059	-0.012	-0.230	-0.015	-0.230	0.041
Living situation	0.005	-0.002	0.025	-0.015	-0.073	0.026	-0.137	0.029
Disease and/or impairment	0.048	0.003	0.067	-0.002	0.058	-0.011	0.097	0.001
Recognition of forgetfulness	-0.147	-0.002	-0.102	-0.009	-0.104	0.001	-0.108	-0.006
Self-rated health	-0.390	0.003	-0.353	-0.012	-0.354	0.001	-0.257	-0.006
Municipality	-0.100	0.019	-0.075	-0.034	-0.166	-0.008	-0.110	0.002
	Volunteer group (ref. never)							
	Twice a week +		Once a week		Once or twice a month		A few times a year	
	Raw	Weighted	Raw	Weighted	Raw	Weighted	Raw	Weighted
Sex	0.084	0.056	0.026	0.053	-0.085	0.006	0.113	0.015
Age	-0.195	-0.053	-0.284	-0.032	-0.235	-0.014	-0.190	-0.037
Years of education	0.306	0.000	0.369	-0.004	0.208	0.018	0.113	0.008
Equivalent income	0.035	0.011	0.122	0.002	0.123	0.009	0.067	0.006
Marital status	-0.089	-0.017	-0.120	-0.006	-0.109	-0.022	-0.188	-0.021
Living situation	0.007	-0.017	0.000	0.016	-0.046	0.012	-0.091	-0.009
Disease and/or impairment	0.149	0.007	0.170	-0.002	0.051	-0.002	0.085	0.007
Recognition of forgetfulness	-0.131	0.006	-0.096	-0.035	-0.138	-0.002	-0.150	-0.009
Self-rated health	-0.367	0.014	-0.322	-0.012	-0.262	-0.027	-0.232	-0.029
Municipality	0.054	-0.008	-0.016	0.022	-0.005	-0.003	0.028	-0.041

	Employment status (ref. retired / never had a job)	
	Employed	
	Raw	Weighted
Sex	0.286	-0.015
Age	-0.500	0.036
Years of education	0.032	-0.003
Equivalent income	0.299	-0.033
Marital status	-0.106	0.031
Living situation	-0.087	0.016
Disease and/or impairment	0.244	-0.003
Recognition of forgetfulness	-0.048	0.002
Self-rated health	-0.260	-0.009
Municipality	-0.002	0.003
