# Supplementary material for: Differences in Cumulative Long-Term Care Costs by Community Activities and Employment: A Prospective Follow-Up Study of Older Japanese Adults
Source: Int J Environ Res Public Health. 2021 May 19;18(10):5414. doi: 10.3390/ijerph18105414 (PMC8158700; doi:10.3390/ijerph18105414)
Supplement: Supplementary file 1 [file ijerph-18-05414-s001.zip › 9_Supplementary Material 3.pdf]

Table S3. C-statistics using a multinomial regression model [1]

	A few times a year	Once or twice a month	Once a week	Twice a week +
Hobby activities group (ref. never)	0.643	0.657	0.682	0.659
Sports group or club (ref. never)	0.714	0.706	0.682	0.651
Volunteer group (ref. never)	0.666	0.631	0.658	0.656

	Employed
Employment status (ref. retired / never had a job)	0.709

1. C statistics by multinominal regression model in order to calculate generalized propensity scores were not high: hobby activities group=.643 to .682, sports group or club=.651 to .714, volunteer group=.631 to 666, employment=.709. However, it is not necessarily to mean undesirable model, because the goal of a propensity score model is to efficiently control confounding, not to predict treatment or exposure.
