# Supplementary material for: Differences in Cumulative Long-Term Care Costs by Community Activities and Employment: A Prospective Follow-Up Study of Older Japanese Adults
Source: Int J Environ Res Public Health. 2021 May 19;18(10):5414. doi: 10.3390/ijerph18105414 (PMC8158700; doi:10.3390/ijerph18105414)
Supplement: Supplementary file 1 [file ijerph-18-05414-s001.zip › 9_Supplementary Material 4.pdf]

Table S4. Robustness checks examining differences in cumulative LTC service cost by community activities and employment status[a)]

	Type of community activities								
	**Hobby activities group**			**Sports group or club**			**Volunteer group**		
	**Model 1** Torbit model	**Model 2** Only survival case	**Model 3** Monthly average cost in survival time	**Model 1** Torbit model	**Model 2** Only survival case	**Model 3** Monthly average cost in survival time	**Model 1** Torbit model	**Model 2** Only survival case	**Model 3** Monthly average cost in survival time
	thousand USD (95% CI)	thousand USD (95% CI)	ten USD per month (95% CI)	thousand USD (95% CI)	thousand USD (95% CI)	ten USD per month (95% CI)	thousand USD (95% CI)	thousand USD (95% CI)	ten USD per month (95% CI)
**Freq. of community activities**									
Never	*ref.*	*ref.*	*ref.*	*ref.*	*ref.*	*ref.*	*ref.*	*ref.*	*ref.*
A few times a year	-0.71**	-0.34	-1.18***	-0.17	0.07	-0.47	-0.67**	-0.37	-1.24***
	(-1.11 to -0.31)	(-0.94 to 0.26)	(-1.78 to -0.58)	(-0.72 to 0.39)	(-0.74 to 0.88)	(-1.31 to 0.38)	(-1.16 to -0.19)	(-1.10 to 0.89)	(-1.75 to -0.72)
Once or twice a month	-0.69***	-0.99***	-1.13***	-0.73*	-0.47	-0.33	-0.67*	-0.55	-1.01**
	(-1.03 to -0.36)	(-1.46 to -0.51)	(-1.69 to -0.58)	(-1.33 to -0.13)	(-1.24 to 0.29)	(-1.24 to 0.57)	(-1.19 to -0.16)	(-1.21 to 0.11)	(-1.61 to -0.40)
Once a week	-0.62**	-0.83**	-1.25***	-1.14***	-1.00**	-1.26***	-0.65	-0.16	-0.23
	(-0.97 to -0.26)	(-1.35 to -0.31)	(-1.81 to -0.69)	(-1.63 to -0.65)	(-1.62 to -0.39)	(-1.79 to -0.73)	(-1.44 to 0.15)	(-1.13 to 0.80)	(-1.52 to 1.06)
Twice a week +	-0.80***	-1.01***	-1.11**	-0.95***	-0.93**	-0.99**	-0.85*	-0.10	-0.63
	(-1.17 to -0.44)	(-1.53 to -0.49)	(-1.78 to -0.43)	(-1.34 to -0.57)	(-1.45 to -0.41)	(-1.56 to -0.42)	(-1.65 to -0.04)	(-1.10 to 0.89)	(-1.68 to 0.41)
**Employment status**									
Retired/never had a job	*ref.*	*ref.*	*ref.*	*ref.*	*ref.*	*ref.*	*ref.*	*ref.*	*ref.*
Employed	-1.46***	-0.45*	-0.68**	-1.38***	-0.43*	-0.75***	-1.26***	-0.39*	-0.61**
	(-1.75 to -1.16)	(-0.82 to -0.07)	(-1.07 to -0.29)	(-1.69 to -1.08)	(-0.81 to -0.06)	(-1.14 to -0.36)	(-1.56 to -0.96)	(-0.77 to -0.02)	(-1.00 to -0.23)

*** p<.001 ** p<.01 * p<.05   1USD≈100JPY

Model 1: The results were from Tobit regression model with inverse probability weighting (IPW) estimation for complete case analysis. To adopt IPW estimation, the generalized propensity scores were calculated using multinominal regression analysis using all previously listed potential confounders: sex, age, years of education, equivalent income, marital status, living situation, disease and/or impairment, recognition of forgetfulness, self- rated health, and municipality.

Model 2: Statistical model is same as "IPW with MI" model in table 2. The results were based on only survival cases in follow-up period.

Model 3: Statistical model is same as "OLS" model in table 2 (complete case analysis). The dependent variable is monthly average cost in survival time.
